# Enhancing Uterine Fibroid Care: Clinician Perspectives on Diagnosis, Disparities, and Strategies for Improving Health Care

**DOI:** 10.1089/whr.2023.0113

**Published:** 2024-03-27

**Authors:** Alfu Laily, Isha Nair, Sophie E. Shank, Cameron Wettschurack, Grace Khamis, Chandler Dykstra, Andrea L. DeMaria, Monica L. Kasting

**Affiliations:** ^1^ Department of Public Health, College of Health and Human Sciences, Purdue University, West Lafayette, Indiana, USA.; ^2^ School of Health Sciences, College of Health and Human Sciences, Purdue University, West Lafayette, Indiana, USA.; ^3^ Marian University College of Osteopathic Medicine, Indianapolis, Indiana, USA.

**Keywords:** clinical care, clinicians, health disparities, qualitative, uterine fibroids, women's health

## Abstract

**Objective:**

To explore clinicians' perspectives on diagnosing, treating, and managing uterine fibroids, identifying gaps and challenges in health care delivery, and offering recommendations for improving care.

**Materials and Methods:**

A qualitative design was used to conduct 14 semistructured interviews with clinicians who treat fibroid patients in central Indiana. Interviews were audio recorded, transcribed verbatim, and analyzed using thematic analysis techniques. Constant comparative analysis was used to identify emergent themes.

**Results:**

Four themes emerged. (1) Lack of patient fibroid awareness: Patients lacked fibroid awareness, leading to challenges in explaining diagnoses and treatment. Misconceptions and emotional distress highlighted the need for better education. (2) Inequities in care and access: Health care disparities affected Black women and rural patients, with transportation, scheduling delays, and financial constraints hindering access. (3) Continuum of care: Clinicians prioritized patient-centered care and shared decision-making, tailoring treatment based on factors like severity, location, size, cost, fertility goals, and recovery time. (4) Coronavirus disease 2019 (COVID-19) impact: The pandemic posed challenges and opportunities, prompting telehealth adoption and consideration of nonsurgical options.

**Conclusions:**

Clinician perspectives noted patient challenges with fibroids, prompting calls for enhanced education, interdisciplinary collaboration, and accessible care to address crucial aspects of fibroid management and improve women's well-being.

**Practice Implications:**

Clinicians identified a lack of patient awareness and unequal access to fibroid care, highlighting the need for improved education and addressing disparities. Findings also emphasized the importance of considering multidimensional aspects of fibroid care and adapting to challenges posed by the COVID-19 pandemic, recommending broader education, affordability, interdisciplinary collaboration, and research for better fibroid health care.

## Introduction

Uterine fibroids—benign tumors of the uterus—account for over $34 billion in annual diagnostic and treatment costs in the United States,^1^ surpassing the annual cost of breast, colon, or ovarian cancers.^2^ Uterine fibroids (hereafter referred to as fibroids) affect 65% of U.S. women and 70%–80% of global women by age 50.^3,4^ Fibroids lead to most gynecologic hospitalizations and hysterectomies in the United States.^5^ Although non-metastatic and commonly asymptomatic, they could manifest into chronic symptoms in up to 50% of reproductive-age women, impacting their quality of life.^4^ Symptomatic patients usually exhibit heavy menstrual bleeding and prolonged menstrual cycles (59%), abdominal pain (74%), constipation (64%), and bladder disruptions (59%).^6^

Risk factors for developing fibroids include early menarche, nulliparity, delayed pregnancy, obesity, hypertension, age, race, and family history.^7–9^ In addition, Black women have an increased risk of fibroid development,^7,9^ and they experience two-to-three times greater incidence and more severe symptoms than White women.^5,10,11^ Black women are also less likely to receive fibroid treatment than White women,^12^ indicating significant health disparities.

Despite the extensive negative impact of fibroids, health care and treatment are often delayed for years due to a lack of awareness.^13^ Almost half of the women at the time of diagnosis were unaware of fibroids and associated health issues.^4^ Limited knowledge of fibroids and normal menstruation may lead women to a false sense of normalcy, delaying disease recognition and health care-seeking behavior.^4^ This lack of awareness prolongs the time to clinical diagnosis, potentially requiring more invasive treatment options—a consequence that cannot only be financially costly but also significantly impact the quality of life.^14,15^ Improving fibroid awareness aligns with patient-centered research to better understand women's experiences, ensuring tailored fibroid treatment and management options with patients' overall needs and desires.^16,17^

Due to the varying presentation of fibroids, clinicians must individualize treatment plans for many factors, including the number, size, and location of fibroids, severity, and patient's goals and preferences for treatment outcomes.^7,18^ Exploring how clinicians diagnose, treat, and manage fibroids is particularly important given recent findings demonstrating a significant reduction in symptoms and increased quality of life following surgical and/or nonsurgical treatment for affected women.^13^ Furthermore, most current research on this topic is limited to quantitative methodology, which does not fully capture participants' experiences and personal narratives. Therefore, this study utilized a qualitative approach to explore clinicians' perspectives on diagnosing, treating, and managing uterine fibroids, identifying gaps and challenges in health care delivery, and offering recommendations for improving patient care.

## Materials and Methods

In this qualitative study, 14 semistructured interviews were conducted with clinicians in central Indiana to capture their experiences in diagnosing and treating fibroid patients, aiming to understand their perspectives on fibroid health care.

### Recruitment

Eligible participants included Obstetrics and Gynecologies (OB/GYNs), family physicians, and fertility specialists practicing in central Indiana, USA. Recruitment occurred from May 2021 to January 2023 through email, the Indiana CTSI mailing list, and peer referrals. Clinicians completed a screening questionnaire and demographic survey. Informed consent, including audio recording permission, was obtained electronically and verbally before interviews. Research protocols were approved by the first author's institutional review board.

### Interviews

We conducted 14 web-based or phone call interviews with clinicians, each lasting ∼30 minutes (31.2 ± 0.4). Four researchers (I.N., S.S., C.K., C.D.) took turns serving as interviewers and notetakers. Three interviews were conducted by a single interviewer, while 11 interviews were conducted with 1 interviewer and 1 notetaker. Semistructured interview guides found in Supplementary S1 were used to provide flexibility and encourage open conversations. The interview topics were meticulously determined through an extensive review of relevant literature and initial discussions with fibroid patients involved in this overarching project.^19^ Interviews covered various topics, including diagnosis, psychological and social impact, treatment, patient education, and health care disparities. These topic selections played a pivotal role in shaping the interview guide, ensuring comprehensive coverage of a diverse range of relevant themes.

It is crucial to note that the emergence of themes during the interviews was not dictated by the preestablished topics; rather, the guide served as a flexible framework to maintain consistency across interviews, allowing participants to share their experiences and insights. Clinicians received a $50 gift card incentive for their participation. We will present quotes from our participants along with the interview identification number and clinical specialty, such as (114-OB/GYN), after each quote.

## Analysis

We utilized thematic analysis and techniques for a constant comparative approach to data analysis.^20,21^ Our thematic analysis approach guided us in identifying, organizing, and analyzing data in multiple systematic phases.^20,22^ After transcribing all interviews verbatim in three rounds (used Otter.AI then followed by two rounds of manual transcriptions), we conducted an immersive data content review to ensure uniform familiarity among researchers.^20^ Then, a “dualistic” deductive/inductive approach was used for codebook development to foster a greater data representation.^23,24^ The codebook book was developed based on interview guides, initial reading of transcripts, and existing literature. We used HyperRESEARCH 4.5.1 to conduct multiple rounds of open and axial coding until saturation was reached.^20,21,25^ A.L. led codebook development, and A.L., I.N., S.S., G.K., and C.K. performed coding. Meetings were held among the coders to ensure interrater reliability.

Coded data were organized into potential themes/subthemes, represented visually in a thematic map, and underwent multistage validation.^20^ Thematic development was data-driven with minimum relation to the original research questions or “analytic preconceptions.”^20,22^ We further “refined and defined” theme content and names to guarantee data accurately and sufficiently portray each theme's “essence” or identity.^20^ We met biweekly to discuss research progress, evaluate thematic development, and engage in “peer debriefing” to ensure that all aspects of data were thoroughly exposed and analyzed.^22^

## Results

### Participant characteristics

In this study, the participants had a mean age of 39.57 years (standard deviation [SD] = 6.37) and a mean of 12.21 years (SD = 6.34) of experience working in the women's health care area. The majority identified as heterosexual/straight (92.8%; *n* = 13). Regarding race and ethnicity, 71.4% were White or Caucasian (*n* = 10), and 85.7% were not Hispanic (*n* = 12). Professionally, 78.6% specialized in OB/GYN (*n* = 11), while other specialties included Family Medicine (7.1%; *n* = 1), Women's Health (7.1%; *n* = 1), and Minimally Invasive GYN Surgery (7.1%; *n* = 1). Clinician types comprised 78.6% physicians (*n* = 11) and 21.4% nurse practitioners (*n* = 3). About 28.6% (*n* = 4) worked in a federally qualified health center. Additional details can be found in Table 1.

**Table 1.** Participant Characteristics

Characteristic	***N*** (%) or Mean ± SD
Age	39.57 ± 6.37
Sexual orientation	
Heterosexual/straight	13 (92.8)
I prefer not to answer	1 (7.1)
Ethnicity	
Not Hispanic or Latina/x	12 (85.7)
I prefer not to answer	1 (7.1)
Other	1 (7.1)
Race	
White or Caucasian	10 (71.4)
Black or African American	3 (21.4)
Asian or Asian American	1 (7.1)
Specialty	
OB/GYN	11 (78.6)
Family medicine	1 (7.1)
Women's health	1 (7.1)
Minimally invasive GYN surgery	1 (7.1)
Clinician type	
Physician (MD, DO, or other)	11 (78.6)
Nurse practitioner	3 (21.4)
Years worked in women's health	12.21 ± 6.34
Federally qualified health center status	
Yes	4 (28.6)
No	8 (57.2)
Unsure	2 (14.2)
Type of health care setting	
Urban	9 (64.3)
Suburban	4 (28.6)
Some other setting	1 (7.1)
Household income	
Comfortable	13 (92.8)
I prefer not to answer	1 (7.1)

OB/GYN, obstetrics and gynecology; SD, standard deviation.

### Themes and exemplar quotes

Data analyses revealed four main themes, each with respective subthemes, as outlined below. Quotes from clinicians, along with their health care specialty, are presented. Additional quotes can be found in Table 2 for reference.

**Table 2.** Participant Additional Quotes

Theme	Subtheme	Exemplar quote
Lack of patient awareness of fibroids	—	I think there's an equal number of women who get sent to me because of asymptomatic fibroids that were diagnosed on imaging, and they had no idea. And some of them are fairly significant. You know, 10, 12, you know, centimeter fibroid, they had no idea [107, OB/GYN]. Patients who have symptoms completely unrelated to the fibroid that had an ultrasound or imaging done in the emergency room or by primary care and they're like, “Oh, you have a, you know, two-centimeter fibroid. Go to gynecology” [105, OB/GYN]. I think the biggest misconception is that they absolutely have to come out. Um, so they're benign, they can stay in there or they can come out it doesn't really matter [104, OB/GYN]. They think it's cancer. They think it's like some form of cancer that's cancer and then that they could like die from it or that they have to get a hysterectomy for it where it's not necessarily the case [113, NP]. They're like, “Aha, this is why I have infertility.” And it probably isn't most of the time. So, I think that's one misconception [105, OB/GYN]. Anytime you mention that there's tumors in the uterus everybody just starts panicking because everybody assumes cancer [110, OB/GYN]. Generally, the patients who are symptomatic is who I give information to [103, NP]. I mean specific information about fibroids is for patients who have the fibroids [110, OB/GYN]. The big challenge is trying, language services. Trying to get an interpreter for a language, and then the Wi-Fi goes out. Or there's only one interpreter available [104, OB/GYN].
Inequities in fibroid-related care and access	—	Because of a language barrier, which is very common, sometimes maybe information isn't relayed 100% [106, OB/GYN]. We have a large Haitian Creole population, but we don't have much resources in that language as far as handouts are explaining things that we would give like our English or Spanish speaking patients [106, OB/GYN]. The majority of big issues with fibroids are African Americans. [109, OB/GYN] We had a larger African American population where we trained, that's definitely the population that's most impacted by fibroids compared to um other races [113, NP] They don't have a care provider down in that county, and that's probably ∼a 40-minute drive [114, OB/GYN]. I have a probably 5%, 10% in travel from outside of the state [102, Minimally Invasive Gynecology Surgery]. We don't have access here to like a what we call a gynecological oncologist, like a GYN oncologist doctor in the community…And a lot of patients in this community tend to have transportation issues. [112, OB/GYN]. Patients have trouble with sometimes follow up and keeping appointments for a variety of reasons, including transportation and inability to take time off work [105, OB/GYN]. We try and make sure that the financial impact just from a health insurance perspective is minimized. What we cannot help with is this loss of income from when they recover from their surgeries [110, OB/GYN]. On average it's probably about three months [for appointment wait]. [110, OB/GYN].
Uterine fibroids continuum of care	Shared decision-making	So, you need to understand, like really, how much does this affect your day to day life, is it a nuisance or not. And then also, you know, based on where the fibroids are located, changes how we might manage them [102, Minimally Invasive Gynecology Surgery]. We'll come up with a treatment plan so my patients, I, you know, I let them decide what you know really what's gonna work for them [101, OB/GYN]. I would probably suggest medication versus putting them through a major surgery just because there just not a good candidate for surgery [113, NP]. If she is seeking fertility, then that dictates the discussion as far as management options [109, OB/GYN]. I will counsel her if she says, Well, I want to wait and see and she has a 20-centimeter fibroid, and she's bleeding a lot and she has anemia. And we will talk this is probably not the best option at this time [109, OB/GYN]. Then how significantly it's been affecting and how long it's been affecting their life [113, NP]. I take a patient centered approach to essentially every patient and personalize everything to the particular patient [104, OB/GYN]. What are their values, and then I listed them all the options available and talk to them about which ones are best for the values that they expressed, but let them know all the options in case they want to do something different [103, NP]. Once we've gone over all the different options, you know, sort of a shared decision-making process with the patient, as to how we want to move forward [102, Minimally Invasive Gynecology Surgery]
	Treatment decision factors	And then also, you know, based on where the fibroids are located, changes how we might manage them [102, Minimally Invasive Gynecology Surgery]. It depends on location of the fibroids and what the presenting symptom is [104, OB/GYN]. How much is it gonna cost? [106, OB/GYN] A big deciding point is the desire for future fertility. And how soon [104, OB/GYN].
	Absence of needed care	Social burden that's a little bit different subject that if you're talking socially, like cost, money, visits and stuff like that, I personally do not go through that aspects [104, OB/GYN]. If there is a problem, where I feel my staff can help, probably I'll refer them to a social worker [109, OB/GYN]. I personally do not get involved in non-medical stuff [109, OB/GYN].
	COVID-19 pandemic and fibroids care	Patients were less likely to come into a medical office for fear of getting COVID. So, they delayed the treatment [112, OB/GYN]. People that weren't able to get in for care that were probably having, you know, symptomatic fibroids, that, that maybe grew bigger for longer because they couldn't get in [111, OB/GYN]. I think using telemedicine more like both for the convenience of like patients and providers…better than not being seen at all [114, OB/GYN]. Pandemic showed that there are other ways to, to handle it um than just surgical options [113, NP]. More medical and conservative options probably given during the pandemic compared to now [113, NP].
Recommendations for fibroid care improvement	Broaden patient education	Public health programs that will discuss fibroids because it's a huge, I mean, abdominal uterine bleeding is the number one reason why women in their early 40s, late 30s present, um, for specific GYN care [110, OB/GYN]. Largest need is education [112, OB/GYN]. It would be great if they were given the information and then saw me. I think that would be much more beneficial if they had the background information on fibroids [104, OB/GYN]. It's education. Because like I said, my patient population, half the patients have never even heard the term [112, OB/GYN].
	Other significant needs	A place that's kind of only focuses on fibroids, like if there were like certain clinics that just do fibroids [103, NP]. The big push has been to try and find ways that are as minimally invasive as possible [110, OB/GYN]. But having child care and elder care and family care and things like that, so that these patients who either have significant symptoms from their fibroids or surgery, can have time to recover. I think that's a huge need [104, OB/GYN]. Offering behavioral health like therapy for that is very important. Infertility is difficult thing for people to handle [105, OB/GYN]. More physician training programs, specifically, obstetrics training programs focus on, I mean, we always focus on medical treatments and what's new and what's out there [112, OB/GYN]. I would say, um, maybe incorporating baseline female health education in school systems [112, OB/GYN].

COVID-19, coronavirus disease 2019; NP, nurse practitioner.

### Lack of patient fibroid awareness

Clinicians stated that most patients do not know what fibroids are and have difficulty grasping their diagnosis because “*it's just a big struggle trying to explain exactly what a fibroid is*.” (110-OB/GYN). One participant described how “*even if they come with a diagnosis of fibroids, sometimes they don't even know what that means*.” (112-OB/GYN). With minimal patient awareness, clinicians usually initiated conversations about fibroids, except where “*the patient has a family history*” (114-OB/GYN). Misunderstandings of fibroid physiology propel the formation of a variety of misconceptions. Interviews gathered misconceptions such as “*the only treatment is surgery*” (109-OB/GYN), “*they're dangerous or life-threatening*” (112-OB/GYN), “*they [always] cause infertility*” (105-OB/GYN), and “*have to take my ovaries out*” (104-OB/GYN). Further challenges were noted when clinicians described the range of treatment options, as one participant reported:

*“I get sent those patients, but they have no symptoms from them. And now trying to explain what they are and also trying to explain why we don't have to do anything and what the natural history of fibroids are. That's when people get a little confused, [they are like] ‘okay, there's a growth in my uterus, but you don't want to do anything about it?*” (110-OB/GYN).

Patients frequently reacted emotionally after their diagnosis, as described by one clinician, “*people generally freak out when I tell them they have fibroids*” (105-OB/GYN). This was attributed to the lack of awareness of what fibroids are and the assumption by the patient that the diagnosis of a fibroid meant a cancer diagnosis, “*everybody just starts panicking because everybody assumes cancer.*” (110-OB/GYN). Language barriers, minimal health literacy, and a low range of information distribution only to fibroid patients challenged communication effectiveness. One participant noted, “*because of a language barrier, which is very common, sometimes maybe information isn't relayed 100%.*” (106-Nurse Practitioner [NP]). Others struggled with delivering full explanations to patients, “*the patient's level of health literacy, [you] don't want to talk to them as if they don't know anything.*” (113-NP).

Clinicians primarily target fibroid-related information for diagnosed patients, rather than providing information to those without diagnosis but at high risk of developing fibroid. One clinician explained, “*specific information about fibroids is for patients who have the fibroids.*” (110-OB/GYN). Lack of fibroid awareness burdens patients misunderstanding their diagnosis, concluding misconceptions, and experiencing emotional distress.

### Inequities in fibroid-related care and access

Clinicians frequently noted difficulties obtaining transportation to appointments: “*A lot of patients in this community tend to have transportation issues. [..] that can make it difficult to provide the best care*” (112-OB/GYN). Most clinicians correctly noted that Black women are disproportionately impacted, “*There is a tendency for African American women to have a higher likelihood or indices for developing fibroids.*” (112-OB/GYN). Clinicians also described patients of lower socioeconomic status as having difficulty affording treatment: “*There's a fair amount of patients who are financially in difficult situations, and sometimes that creates an issue primarily with being able to afford medications*” (107-OB/GYN).

Clinicians noted scheduling delays for appointments, with one reporting that it could be “*upwards of two to three months out depending on if they're new*” (113-NP), while another said a patient could be seen “*probably within a month if they're established.*” (111-OB/GYN). Even for patients who can see their clinicians, there are often delays in care due to long wait times for test results because health systems are “*pretty behind on imaging.*” (105-OB/GYN).

This problem is exacerbated in rural communities as one clinician noted that “*because there's a small community, sometimes there's a higher demand for us than we're able to supply*.” (113-NP). One participant mentioned that patients travel farther to their clinic for financial benefits, saying “*[patients] come to [my hospital] because of how we do our insurance and put them on a sliding scale based on their income.*” (106-NP). Financial concerns were commonly mentioned, with some clinicians noting social workers to assist patients, “*we try and make sure that the financial impact just from a health insurance perspective is minimized.*” (110-OB/GYN). This participant continued to mention costs outside their control, noting the “*loss of income from when they recover from their surgeries.*” (110-OB/GYN). These health care system components challenge many patients trying to access fundamental fibroid care.

### Uterine fibroid continuum of care

#### Shared decision-making

Clinicians used “*a patient-centered approach to essentially every patient and personalize everything to the particular patient.*” (104-OB/GYN). Clinicians emphasized that the treatment option had “*to be a mutual agreement.*” (109-OB/GYN), and that they would “*have a little bit more detailed discussion that probably [the patient] may need to consider [a different treatment option]*” (109-OB/GYN). Many perceived their patients as satisfied with the information received for their decision-making: “*I haven't got any complaints so far that anybody is not happy with the information we provide*” (109*-*OB/GYN). In contrast, others noted dissatisfaction when patients have “*an adverse, like a side effect*” (106-NP), which was common. At times, patients “*just switched doctors [because] they decided they don't want to see me*” (101-OB/GYN). Overall, shared decision-making was emphasized to provide the best possible care for patients.

#### Treatment decision factors

Clinicians mentioned that the fibroids' severity, location, and size influenced treatment discussions, stating “*it depends on patient, patient desires, plus where they are in terms of like, how severe their diagnosis is.*” (113-NP) and “*tell a patient about every treatment out there that I know of for fibroids. And then we kind of funnel down to, based on your fibroids and their size and their location.*” (102-Minimally Invasive Gynecologic Surgeon [MIGS]). Cost was often cited as a reason a patient would opt for a certain treatment plan: “*a lot of patients are like, ‘well, how much is it gonna cost?*” (106-NP).

Fertility and family planning goals also influenced treatment decisions, with one clinician noting “*if she is seeking fertility, then that dictates the discussion as far as management options.*” (109-OB/GYN). Patients preferred treatments with shorter recovery times to return to “normal” more quickly. Common patient questions related to “*the recovery time period, the access to minimally invasive surgical procedures that will reduce recovery length.*” (110-OB/GYN).

#### Absence of needed care

Even though clinicians mentioned various symptoms caused by fibroids that impact quality of life, several stated that they had no reliable approach to address the multidimensional (*i.e.,* social, emotional, mental) aspects of fibroid care. In addition to being costly, fibroids impact everyday activities:

*“Those who have heavy bleeding [..] have issues with being at work or out, you know, doing their daily activities and bleeding through their clothing, and having to go home and change, can be disruptive to their sex lives*.” (102-MIGS).

Despite these impacts, when clinicians were asked if they address multidimensional aspects of fibroids, many offered none: “*Oh, I would say zero*” (104-OB/GYN). Some noted it was uncommon to provide this type of care, but when it was needed, they would give a referral: “*I haven't been involved in a lot of those cases, I can ask my staff to help the patient through a social service worker*” (109-OB/GYN). Clinicians reveal further ways to improve care by focusing more on the multidimensional aspects of fibroids.

#### Coronavirus disease 2019 pandemic and fibroid care

The coronavirus disease 2019 (COVID-19) pandemic challenged how patients sought care and their interest in treatment options. One clinician recounted how a patient declined surgery due to mandatory presurgery COVID testing because they believed that the “*COVID test would give them COVID*” (113-NP). Clinicians also recalled issues with staffing, such as “*having enough staff, healthy and available, to be able to see enough patients.*” (112-OB/GYN).

Alternatively, the pandemic did lead to some positive changes in fibroid care, including telehealth, which many clinicians spoke of highly. As one explained, it allowed them to “ac*tually reach some of these patients who we otherwise, maybe weren't able to reach pre-pandemic*” (102-MIGS). Clinicians felt the pandemic exposed alternatives to handle fibroids, pushing patients and clinicians to consider “*medical and non-medical options [as they were] less apt to jump into surgical options*” (113-NP). The pandemic has had a multifaceted impact on fibroid care.

### Recommendations for fibroid care improvement

#### Broaden patient education

Clinicians mentioned “*half the patients have never even heard the term [fibroids]*” (112, OB/GYN), and recommended, “*I would love it if they had [fibroid] information available to patients on healthcare system websites. So: 1) it could be reputable and 2) easily accessible to anyone who had questions*” (102-MIGS). One clinician described a preference for fibroid education during routine gynecologic care visits for all women, “*in well woman visits, touch on topics that affect women just so they know when to come in to see us.*” (113-NP). Finally, one clinician offered up how other fields should get involved, “*public health programs [should] discuss fibroids*” (110-OB/GYN), noting an opportunity for population-based education to broaden awareness.

#### Other significant needs

Fibroids affect patients “*certainly financially, you know, here's a patient who's missing work already because of the symptoms.*” (102-MIGS). For this reason, clinicians advocated that fibroid care be more affordable and that “*some of the stuff that could be cheaper, less expensive.*” (101-OB/GYN). Furthermore, clinicians endorsed expanding insurance coverage of treatment options, saying “*this medication [..] sounds great. But it's not covered by insurance. And a lot of patients ask for it. I think coverage of those medications will be the most important thing.*” (113-NP).

Clinicians advocated for better collaboration, as fibroid care needs a broader scope of specialist partnerships. One clinician stated, “*I feel like fibroids sometimes takes multiple specialties, so having some of those networks pre-made […] could be helpful.*” (103-OB/GYN). Moreover, they promoted the importance of continuing research on fibroid prevention, as one questioned “*how to prevent women from even developing them in the first place*” (102-MIGS), and urged finding more minimally invasive treatments, claiming “*the big push has been to try and find ways that are as minimally invasive as possible.*” (110-OB/GYN). Moving forward, clinicians advocated for more affordable care, further fibroid research, and improved dissemination.

## Discussion

We explored fibroid health care delivery by conducting 14 interviews with Indiana-based clinicians, uncovering gaps in diagnosis, management, and patient support. Education and awareness emerged as critical factors for improving fibroid care, addressing the substantial impact on patients' well-being. Addressing fibroid care is crucial to ensuring women's well-being, reducing health care disparities, and enhancing overall health care access.

Despite the high prevalence of fibroids, clinicians observed low awareness of fibroids before diagnosis. Our findings suggested that some clinicians prefer distributing fibroid information exclusively to diagnosed patients, which may contribute to low fibroid awareness, highlighting the potential benefit of adopting an approach to provide fibroid information to all women during routine gynecological visits, as recommended by some other clinicians in our study.

This lack of patient awareness challenged clinicians to guide management options, predominantly when patients were asymptomatic and required minimal or no intervention. While “watchful waiting” was suitable for asymptomatic patients without immediate treatment needs,^26^ clinicians reported that their patients were dissatisfied and preferred more active intervention. Moreover, clinicians voiced that women struggled to comprehend the term “benign,” often mistaking it for cancerous, which aligns with previous work,^27^ suggesting the need for improved terminology during initial conversations. To bridge this gap, clinician–patient communication and education should be fostered to avoid negative care experiences potentially affecting care engagement.^19,28^

Clinicians reported several health disparities in fibroid care, resulting in delayed treatment and poor health outcomes. Our clinicians noted that Black women experienced worse health outcomes than their White counterparts, potentially due to delayed care seeking, aligning with previous research.^7,9^ Concerns were also raised about rural patients lacking access to specialists, leading to longer travel and wait times, deterring timely treatment. In 24 out of 92 Indiana counties, OB/GYN services were absent,^29^ indicating that women in rural areas must travel longer to reach clinicians with the necessary expertise and resources for fibroid treatment. In addition, clinicians voiced that high demand for care did not match clinician availability, resulting in longer waits and missed appointments in rural areas. To address disparities between rural and urban areas, targeted efforts are needed to improve access to specialist services in underserved regions, like incentives for rural specialists and remote consultations,^30,31^ supporting equitable access to fibroid care.

Despite clinicians recognizing the adverse impact of fibroid symptoms on patients' quality of life, they provided minimal or no resources to address financial, psychological, or social issues arising from the condition. Clinicians acknowledged that patients suffer financial-related issues, such as difficulty affording medications and unpaid work-leave, preventing timely and routine care. Furthermore, psychosocial issues from fibroid symptoms also disrupt daily life (*e.g.,* stress from sex-life disruptions, embarrassment due to heavy bleeding). However, they felt addressing anything beyond the physical aspects of fibroids was beyond their scope of practice. Although some referred patients to social workers for these types of support, this approach was not universal. This might also contribute to urban–rural disparities mentioned previously due to inadequately staffed social workers in rural compared to urban clinics.

These findings suggest the necessity for integrated health services to meet patients' needs, which should be arranged within women's health services.^32^ Recent studies showed that a multidisciplinary health service combining gynecology and psychology significantly impacted women's mental health.^33,34^ Therefore, it is crucial to implement integrated care as a standard practice in women's health, which can be achieved by incorporating case managers in clinics to guide and support patients in accessing comprehensive resources^34^ and/or implementing artificial intelligence to seamlessly bridge multiple specialists to treat multimorbid patients (*e.g.,* OB/GYN patient with psychiatric comorbidities).^35^

Clinicians further endorsed the importance of having a well-established specialist network to streamline patient referrals. Patients could benefit from the collaboration of multidisciplinary specialists, such as OB/GYNs for choosing treatment, hematologists for addressing heavy bleeding, and surgeons.^36,37^ The absence of such a network could result in fibroid fragmented management and longer referral and consultation wait times,^38,39^ potentially compromising patients' outcome and satisfaction. Studies indicated that a fibroid center with multidisciplinary specialists network could result in increased referral and shorter wait times^38^ and satisfaction of patients for thorough consultations.^40^

Our clinicians employed patient-centered approaches to tailor fibroid care based on patients' intrinsic values, concerns, and expectations. Like previous research, clinicians guided patients in selecting treatment options by presenting all available choices, along with their benefits and risks.^41^ Individualized treatment plans were then developed, considering patient-driven factors such as disease severity, fibroid location/size, treatment cost, side effects, and recovery, similar to what is reported in the existing literature.^7,18^ However, in some cases, patients' preferences were incompatible with their condition, leading to dissatisfaction despite additional counseling provided by clinicians. This highlights the importance of engaging patients in shared decision-making, ensuring that they are fully informed about the costs, benefits, and risks of fibroid treatment options; even if patients' original preferences may not always align with the final decision.^42^ Clinicians should aim to validate patients' choices to enhance satisfaction.^28^

While treatment plans cater to individual patient needs, clinicians also aim to customize in-clinic patient education using handouts and online resources. Like other studies, our research participants advocated for integrating fibroid education into routine gynecological visits and raising awareness about fibroids as a public health initiative.^4,43^ This is crucial due to the similarity of fibroid symptoms with typical menstrual symptoms,^44^ and such education can aid in symptom recognition and promote early diagnosis. To achieve public awareness, distributing pamphlets in retail settings and conducting educational campaigns at high schools have been suggested.^19^

In addition, to adapt to a rapidly changing scientific landscape, clinicians should receive continued education on fibroids,^45^ including information on new treatment modalities, especially minimally invasive treatments as clinicians noted that patients demanded shorter recovery time. Nonsurgical treatment options (*e.g.,* uterine artery embolization) promisingly decreased recovery time,^46^ while producing comparable success in terms of improving quality of life.^47^ Therefore, by staying informed about these advancements through continued education, clinicians can effectively offer nonsurgical treatment options to their patients, leading to improved patient satisfaction and quality of life.

Clinicians referenced that barriers to fibroid care were exacerbated during the COVID-19 pandemic, resulting in a shortage of clinicians and increased patient hesitancy to seek treatment, consistent with recent gynecologic studies.^48,49^ However, clinicians identified that the pandemic also brought about long-term solutions, such as the increased use of telehealth and the tendency to opt for nonsurgical treatment. The pandemic led to a greater reliance on electronic services (*e.g.,* telehealth) to reach more previously inaccessible patients and forced clinicians to explore less invasive and immediate treatment options.^50,51^ Research shows a growing trend of patients using complementary and alternative medicine (CAM) to manage fibroid symptoms,^52–54^ suggesting that clinicians should be aware of CAM's role in fibroid management and consider it as a potential treatment option. Ultimately, the pandemic revealed the importance of clinicians' resilience in providing quality health care in a continuously evolving world.

This study is one of the first to use qualitative methodology to understand fibroid health care delivery and identify areas for improvement from clinicians' perspectives. However, there are some limitations. All participants who expressed willingness to be interviewed were selected, introducing a potential selection bias. Although the study initially aimed for 20 participants, it had to conclude at 14 due to recruitment challenges, resulting in a smaller sample size. However, data saturation was still achieved. It is important to note a potential over-representation of academic clinicians, given that the recruitment used an academic-hospital network mailing list. The recruitment process was limited to Indiana clinicians in the central region, which may reduce the generalizability of the findings to other regions.

Qualitative research is prone to recall and social desirability biases, although efforts were made to minimize these biases, such as ensuring confidentiality. Our somewhat homogenous sample (predominantly non-Hispanic White, OB/GYN specialists, urban settings) might limit the generalizability of findings to other populations and regions. Including a more diverse range of clinicians, such as those from different specialties, ethnic backgrounds, and geographical areas, could enhance future studies' applicability to a broader context. Our guide did not explicitly prompt individual clinicians to provide specific solutions for the identified issues, resulting in a lack of clinician recommendations. Furthermore, COVID-19-related questions were added during data collection after the first few interviews, limiting the comprehensive insights of all participants.

## Conclusions

This study's findings shed light on the challenges patients face with fibroids, as seen through the eyes of clinicians. The pandemic prompted both hurdles and progress in fibroid health care. Drawing from their extensive experience, clinicians pinpoint key areas for enhancing fibroid care, advocating for improved patient and clinician education, interdisciplinary teamwork, affordable insurance, and accessible care for all affected individuals. By addressing these needs, we can work toward practical solutions that significantly improve the quality of life for women navigating the complexities of fibroids.

### Practice implications

Our study's findings have significant implications for enhancing uterine fibroid health care strategies. Clinicians noted a substantial lack of patient awareness, leading to misunderstandings and emotional distress, alongside prevalent misconceptions about fibroid characteristics. Disparities in health care delivery, exacerbated by transportation issues and financial constraints, highlighted the urgent need for addressing inequities. While clinicians emphasized shared decision-making based on factors like fibroid severity, location, cost, and recovery time, there was a tendency to overlook the multidimensional aspects of fibroid care, including social, emotional, and mental dimensions.

The COVID-19 pandemic prompted adaptations, such as the increased use of telehealth, revealing gaps in patient understanding and access. Moving forward, strategies for improvement should include comprehensive patient education, increased affordability of treatments, expanded insurance coverage, interdisciplinary collaboration, research on prevention, and the pursuit of minimally invasive options. These findings showcase the necessity for a paradigm shift in clinical standards, emphasizing a patient-centered, multidisciplinary, and adaptable approach to uterine fibroid health care.

## Ethics Approval and Consent to Participate

This study was approved by the Purdue Institutional Review Board (IRB-2021-365). All study participants provided written and verbal informed consent, including consent to be audio recorded.

## Supplementary Material

Supplemental data

## Abbreviations Used

CAM: complementary and alternative medicine
COVID-19: coronavirus disease 2019
MIGS: Minimally Invasive Gynecologic Surgeon
NP: nurse practitioner
OB/GYN: obstetrics and gynecology
SD: standard deviation
