# Supplementary material for: Enhancing Uterine Fibroid Care: Clinician Perspectives on Diagnosis, Disparities, and Strategies for Improving Health Care
Source: Womens Health Rep (New Rochelle). 2024 Mar 27;5(1):293–304. doi: 10.1089/whr.2023.0113 (PMC10979696; doi:10.1089/whr.2023.0113)
Supplement: Supplemental data [file Supp_DataS1.docx]

**Clinian Interview Guide**

**Improving Overall Quality of Life for Uterine Fibroid Patients**

PI: Andrea L. DeMaria, PhD, MS; Department of Public Health, College of Health and Human Sciences; Purdue University; West Lafayette, IN; USA; [ademaria@purdue.edu](mailto:ademaria@purdue.edu); Co-I: Monica L. Kasting, PhD; Department of Public Health, College of Health and Human Sciences; Purdue University; West Lafayette, IN; USA; [mlkastin@purdue.ed](mailto:mlkastin@purdue.ed)

Welcome. I want to start by saying how thankful I am that you are here today to help me with this research project I am conducting with my research team about uterine fibroids.

My name is ________________ and I will be conducting this research interview. I am part of an interdisciplinary team at Purdue University conducting research on women’s health issues, including uterine fibroids. [Optional if note taker is present: This is_________ and she will be taking notes so that I can completely concentrate on the interview and what you are saying during our time together.]

First, I want to thank you for completing the electronic consent form and participant survey. As a brief reminder, the goal of the study is to examine the healthcare experiences of uterine fibroids patients across the continuum of care, from early symptoms through diagnosis, treatment, and management.

Your experiences as a healthcare provider of uterine fibroid patients are important to me. As the interviewer, I will ask you questions related to your experiences with diagnosing and treating uterine fibroid patients. As the participant, you are encouraged to share experiences you feel comfortable with, which will be kept completely anonymous. I want you to know that your opinions are very important and there are no right or wrong answers. You do not have to answer all questions, you can ask me to skip a question, and you can end the interview at any time. And if you need clarification on any question, please do not hesitate to ask me to further explain.

As was indicated in the consent form, I will be audio recording today’s conversation because I do not want to miss any of your comments. But, your name will always remain confidential, so anything we discuss today will not be tied to your name in any final reports or publications.

My role today is to ask questions and listen. I will be moving the discussion from one question to the next. During our talk, I may sometimes have to interrupt you if I feel we are running out of time and there are several more questions to get to; please do not feel this has to do with what you are saying.

Do you have any questions before we get started? [wait a few moments]

Ok, great! I will now begin the audio recording. From here on forth, both of our voices will be captured.

**General Rapport Building: First I would like to ask some general questions about your healthcare expertise and the community you work in.** (Spend approximately 5 minutes on this section.)

1. How would you describe in brief detail your area of expertise? **[Following questions will be adjusted to their specific field/expertise]**

*Probe:* How long have you been working in this field?

1. How would you describe the community you work in?

*Probe*: What are the general demographics of the community you work in? In terms of socioeconomic status, insurance age, race/ethnicity, etc.

*Probe:* What are some positive attributes of the community you work in? Why?

*Probe*: What are some of the challenges of working in this community? Why?

*Probe*: Do you think your patients would identify similar things? Why or why not?

1. Walk me through a typical routine visit for a new patient.

*Probe*: In general, what types of questions do you ask?

*Probe:* How do most patients feel during a typical visit, from your perspective? Nervous, withdrawn, open, curious?

*Probe*: How do you determine the comfort level of your patient? What do you do to increase their comfort during the consultation?

**UTERINE FIBROIDS: Next I would like to ask some questions specifically related to uterine fibroid diagnosis, care, and treatment.** (Spend approximately 35 minutes on this section.)

1. Tell me about a typical clinical presentation for uterine fibroids.

*Probe:* What types of symptoms do you typically see in patients who have uterine fibroids?

*Probe:* Who initiates conversations about uterine fibroids? The patient? A family member? You?

*Probe:* How do patients typically find out they have uterine fibroids?

*Probe:* How do patients typically react when you tell them they have uterine fibroids?

*Probe:* What populations are the most impacted in your community by uterine fibroids? Or, in other words, how would you describe the population you serve in terms of uterine fibroid's treatment?

*Probe:* How does uterine fibroids affect your patient’s lives?

1. Tell me about the type of information you provide patients about uterine fibroids.

*Probe*: What are the common misconceptions about uterine fibroids that you address?

*Probe:* What type of information do patients typically bring with them about uterine fibroids?

*Probe:* Where do patients find information or misinformation about uterine fibroids?

*Probe:* What kind of educational information does your clinic provide to the patients to educate them about uterine fibroids? What kind of information does it entail?

*Probe:* What kind of educational information does your clinic provide to patients to educate them or support them with the emotional and social aspects of dealing with uterine fibroids? Are there multiple media types?

*Probe:* In your opinion, is the information provided understandable to patients?

*Probe:* What kinds of questions do you typically receive when communicating this information?

*Probe:* When do you think is the ideal time to provide this information?

*Probe:* Are there barriers to access information about uterine fibroids that you are aware of?

1. How do you distribute the uterine fibroid information?

*Probe:* Who is this information provided to? All patients? Patients who are symptomatic? Patients who have uterine fibroids? Patients with a family history?

*Probe:* How do you decide when to provide this information? Specific factors that influence your decision?

*Probe:* Do you believe the method of information distribution is acceptable to patients? Effective? If no, why not?

*Probe:* If you could distribute information in any way you want, how would you do it?

1. How do you decide which treatments to provide?

*Probe:* What types of factors influence your decision? Physical symptoms? Psychological effects? Side effects?

*Probe:* How involved are patients in making this decision?

*Probe:* With the patients you see, what are the factors that influence the final decision they make about treatment?

*Probe:* Tell me about a time when a patient was unhappy with their treatment decision. Does that happen often?

*Probe:* How much in agreement are you with the treatment decisions patients make?

1. How did the COVID pandemic impact your ability to care for fibroids patients?

*Probe:* What were some challenges you faced? What were some positive aspects that were noted?

*Probe:* What changes did you notice in how your patients managed their fibroids symptoms?

*Probe:* What lessons from the pandemic can be used to develop future fibroids education? Care standards? Policies?

1. Do your patients typically live in the area near your clinic or do they have to travel far to seek treatment?

*Probe:* On average, how far would you say your patients travel to receive the care you provide?

*Probe:* What differences do you notice among your patients who have shorter travel times, to those that have longer travel times?

*Probe:* In what ways do you offer care to hard-to-reach patients? Telehealth?

*Probe:* How long does a patient typically have to wait to get an appointment to see you? For both an established patient and a new patient.

1. When would it be most convenient to incorporate uterine fibroids patient education into your regular clinical care protocol?

*Probe:* Tell me a bit about how you have used a patient-centered approach to uterine fibroids treatment and management.

*Probe:* How can interventions be developed to treat the multidimensional aspects of health impacted by uterine fibroids (for example, incorporating the psychological, social, and financial effects of treatment)?

*Probe:* What do you think the largest need is in terms of providing uterine fibroid diagnosis or treatment?

*Probe:* What would you like to see done to improve uterine fibroid care, from diagnosis through treatment and management?

*Probe:* What do you think is the best way of implementing these improvements to uterine fibroid care?

Those are all the questions I have. I appreciate your time in speaking with me, and your openness and honesty in sharing your professional experience. Are there any final comments you would like to share with me before we conclude our interview?

Ok, I am now ending the audio recording. Anything you say from here on will not be captured.

Thank you for participating today. Your insights are valuable for the success of our research and we appreciate your time. **You should receive your electronic gift card via email within the coming days.**

As a reminder, the study investigator contact information is on your consent form. Please do not hesitate to reach out with any questions, or if you would like to be connected to the study website that will house preliminary results.

Please feel free to pass my contact information along to anyone who might be interested in participating in this study.

Thank you and have a great day!
